# Repairing Bilateral Coronary-Pulmonary Artery Fistulas During Coronary Artery Bypass Grafting

**DOI:** 10.1016/j.atssr.2024.03.002

**Published:** 2024-03-29

**Authors:** Alexander L. Chen, Alessandro Vivacqua, Jeffrey M. Altshuler, Francis L. Shannon, Rajesh C. Gulati, Thomas A. Schwann, Bogdan A. Kindzelski

**Affiliations:** 1 Department of Cardiovascular Surgery, Corewell East William Beaumont University Hospital, Royal Oak, Michigan; 2 Department of Cardiovascular Medicine, Corewell East William Beaumont University Hospital, Royal Oak, Michigan

## Abstract

Coronary-pulmonary artery fistulas (CPAFs) are rare entities that can cause significant left-to-right shunting and complicate routine coronary artery bypass grafting. There are no best practice guidelines and a scarcity of reports regarding concomitant treatment of CPAF with coronary artery disease. We present a case of bilateral CPAFs in a 60-year-old man with symptomatic coronary artery disease treated successfully with coronary artery bypass, epicardial ligation, and transpulmonary closure of CPAF with patch reconstruction. This case highlights the importance of optimal myocardial protection and complete closure of the fistula to prevent risk of coronary steal.

Coronary artery fistulas are rare congenital connections that form between coronary arteries and other cardiovascular structures.^1^ Coronary artery fistulas are rare, making up 0.1% to 0.4% of all congenital cardiac anomalies.^2^^,^^3^ Of these, 15% to 20% are coronary-pulmonary artery fistulas (CPAFs).^4^ Not surprisingly, there is a paucity of reports on optimal treatment strategies for these patients, especially when associated with concomitant severe coronary artery disease. We present a successful surgical approach to managing symptomatic severe 2-vessel coronary disease with multiple CPAFs arising from bilateral coronary arteries.

A 60-year-old man presented to his cardiologist after an elevated cardiac calcium score was discovered. He reported complaints of intermittent chest pain with exertion, which prompted a further ischemic workup. After the initial results, he underwent a coronary angiogram, which revealed the presence of multivessel disease involving 90% stenosis of the proximal left anterior descending artery and 90% midstenosis of the right coronary artery. Supplemental Video 1 and Figure 1A and Supplemental Video 2 and Figure 1B show bilateral CPAFs originating from the left anterior descending artery and the right coronary artery, respectively.

**Figure 1:**
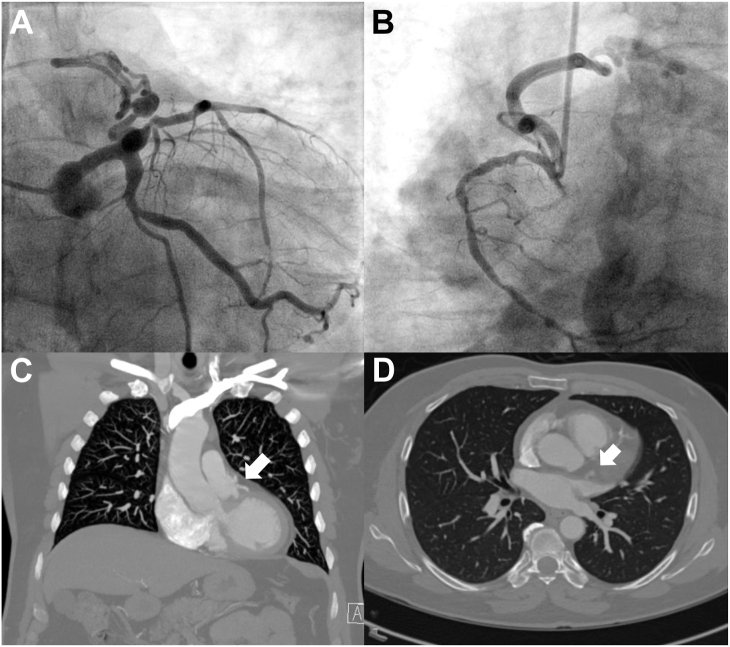
Left coronary angiogram demonstrates fistula formation between the (A) left anterior descending coronary artery and pulmonary artery and (B) fistula formation between the right coronary artery and pulmonary artery. Computed tomography shows fistula feeding into the main pulmonary artery (arrows) with representative (C) coronal and (D) axial images.

The contribution to the ischemic burden of the atherosclerotic occlusive lesion and of the potential coronary steal through the CPAF was unclear. To better understand the fistulous lesions, a coronary computed tomographic (CT) angiogram was performed, confirming the initial findings (Figures 1C, 1D). He was then referred to cardiac surgery for definitive care.

Given his symptomatic multivessel disease and CPAF, the patient was taken for coronary artery bypass grafting and surgical closure of the fistulas. A midline sternotomy and skeletonized left internal mammary artery harvesting were performed. Cardiopulmonary bypass (CPB) was initiated with cannulation of the distal aortic and right atrial appendage. Antegrade as well as retrograde (due to concern of shunting with solely antegrade cardioplegia) blood cardioplegia was used to arrest the heart and was given intermittently thereafter. Bypasses were then performed with the left internal mammary artery used for the left anterior descending artery, and a saphenous vein graft used for the posterior descending artery anastomosis.

Once the bypass grafts were completed, the most superficial CPAF was identified. Epicardial ligation was performed using a 5-0 polypropylene suture. The pulmonary artery was then explored through an incision based on the preoperative CT imaging revealing multiple endoluminal fistulous openings. Additional antegrade cardioplegia was administered, demonstrating additional flow through a small ostium, indicating a fistulous tract opening the main pulmonary artery. This was closed internally using a 6-0 polypropylene suture. The pulmonary artery was then closed using bovine pericardial patch augmentation to avoid unnecessary tension, as suggested in Figure 2. The operation was completed routinely without incident. Total CPB time lasted 90 minutes.

**Figure 2:**
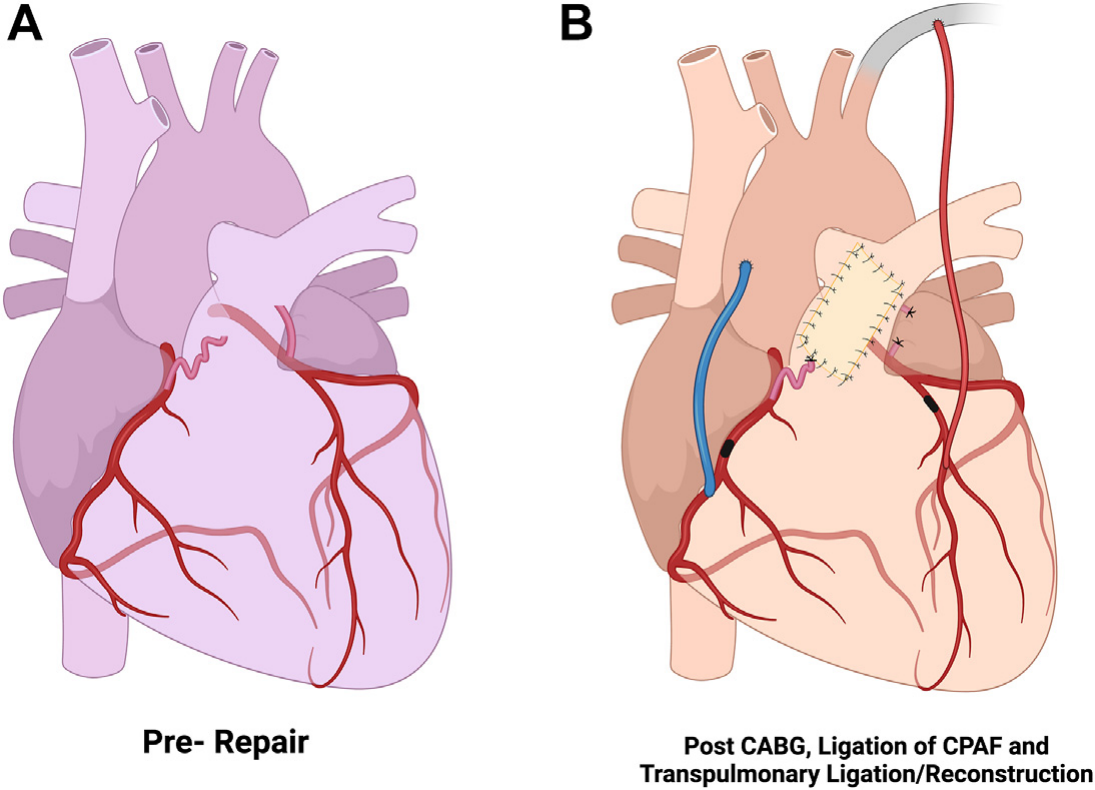
(A) Before the repair. (B) After coronary artery bypass grafting (CABG), pulmonary artery (PA) patch angioplasty, and ligation of coronary-to-PA fistula (CPAF). Created with BioRender.com.

The remainder of the patient’s postoperative course was uneventful, and he was discharged on postoperative day 5. At the 3-month follow-up, the patient was recovering well, without any significant reported issues. Specifically, he reported no further chest pain.

## Comment

Given their infrequency, there is little consensus on the management of CPAF and their natural history. Current guidelines released by the American College of Cardiology and the American Heart Association in 2018 reported no agreement in the management of CPAF.^2^ With advancements in endovascular techniques, case reports of transcatheter approaches have been described with documented success.^5^ However, endovascular interventions are limited when fistulas present with extreme tortuosity and aneurysmal formation.^3^ Although these techniques are available, long-term outcomes still need to be determined.

Surgical ligation has traditionally been the mainstay of therapy, and stand-alone surgical fistula closures are very rare. CPAFs are typically addressed in the setting of comorbid cardiac disease to prevent future complications attributable to the CPAF. The most common surgical techniques described involve direct epicardial ligations and transpulmonary artery closure.^5^ In some instances, coronary artery fistulas result in the formation of aneurysmal segments necessitating complex reconstruction given their increased risk of rupture.^3^^,^^6^ Because many of these are repaired simultaneously with other cardiac comorbidities, CPB is used in most, but not all, of the procedures. Given the rarity of coronary artery fistulas, no randomized data comparing repair methods are currently available or likely forthcoming.

As with most cardiac surgical cases, optimal cardioplegic myocardial protection is crucial. Abnormal cardioplegia drainage through the CPAF away from the affected downstream coronary vessel can make it challenging to ensure optimal cardiac arrest.^3^ Retrograde cardioplegia should be considered to compensate for such aberrant drainage.

To provide a thorough examination and prevent any residual shunting, exploration of the pulmonary artery under CPB has been recommended.^4^ Endoluminal exploration allows for the identification of any additional ostia within the pulmonary artery that may not be visible externally. A thorough examination is necessary to prevent residual anomalous fistulized connections that could lead to inadequate repair.

The most common complication reported with CPAF repair is myocardial infarction (6.5%-11%).^2^^,^^7^ This complication highlights the importance of determining the coronary artery dominance before ligation to prevent potential malperfusion from inadvertent ligation of important distal branches. When coronary artery bypasses are performed concomitantly, ligating all CPAFs is essential to avoid coronary steal and its subsequent detrimental effects.^5^^,^^7^ Long-term outcomes after surgical repair appear favorable, with no recurrence appreciated among multiple studies,^6^^,^^8^ and long-term survival is reported to be 74% successful at 5 years.^6^

In our case, due to the complexity presented by multiple fistulas from 2 distinct coronary arteries, multiple operative techniques were used. Retrograde and antegrade cardioplegia was administered to ensure adequate cardiac protection due to the potential flow diversion from myocardial microcirculation through these fistulas. As with most CPAFs, epicardial ligation was performed of the visible epicardial fistula. In addition, given their subepicardial course, we used an endoluminal closure within the pulmonary artery of the contralateral fistula. This approach allowed ligation of the fistula without added local trauma caused by further dissection.

CPAFs are a rare and unique cardiac anomaly that currently lacks clinical guidelines for management. Surgical approaches will need to be individualized based on specific anatomic considerations and may frequently require more than a simple ligation, especially if multiple fistulas are present. Further investigation is needed to determine the best management strategy for those with CPAFs and concomitant coronary disease.
